# Persistence of chloroquine resistance alleles in malaria endemic countries: a systematic review of burden and risk factors

**DOI:** 10.1186/s12936-019-2716-z

**Published:** 2019-03-12

**Authors:** Moses Ocan, Dickens Akena, Sam Nsobya, Moses R. Kamya, Richard Senono, Alison Annet Kinengyere, Ekwaro A. Obuku

**Affiliations:** 1 Department of Pharmacology & Therapeutics, Makerere University, P.O. Box 7072, Kampala, Uganda; 2 Department of Psychiatry, Makerere University, P.O. Box 7072, Kampala, Uganda; 3 Department of Medical Microbiology, Makerere University, P.O. Box 7072, Kampala, Uganda; 4 Department of Medicine, Makerere University, P.O. Box 7072, Kampala, Uganda; 5 Infectious Disease Institute, Makerere University, P. O. Box 22418, Kampala, Uganda; 6 Albert Cook Library, Makerere University, P.O. Box 7072, Kampala, Uganda; 7 Clinical Epidemiology Unit, Department of Medicine, Makerere University, P.O. Box 7072, Kampala, Uganda; 8 Africa Centre for Systematic Reviews and Knowledge Translation, Makerere University College of Health Sciences, P.O. Box 7072, Kampala, Uganda; 9 Faculty of Epidemiology and Population Health, London School of Hygiene and Tropical Medicine, London, UK

**Keywords:** Chloroquine, Re-emergence, Sensitivity, Plasmodium falciparum, Policy

## Abstract

**Background:**

Chloroquine, a previous highly efficacious, easy to use and affordable anti-malarial agent was withdrawn from malaria endemic regions due to high levels of resistance. This review collated evidence from published-reviewed articles to establish prevalence of *Pfcrt* 76T and *Pfmdr*-*1* 86Y alleles in malaria affected countries following official discontinuation of chloroquine use.

**Methods:**

A review protocol was developed, registered in PROSPERO (#CRD42018083957) and published in a peer-reviewed journal. Article search was done in PubMed, Scopus, Lilacs/Vhl and Embase databases by two experienced librarians (AK, RS) for the period 1990-to-Febuary 2018. Mesh terms and Boolean operators (AND, OR) were used. Data extraction form was designed in Excel spread sheet 2007. Data extraction was done by three reviewers (NL, BB and MO), discrepancies were resolved by discussion. Random effects analysis was done in Open Meta Analyst software. Heterogeneity was established using I^2^-statistic.

**Results:**

A total of 4721 citations were retrieved from article search (Pubmed = 361, Lilac/vhl = 28, Science Direct = 944, Scopus = 3388). Additional targeted search resulted in three (03) eligible articles. After removal of duplicates (n = 523) and screening, 38 articles were included in the final review. Average genotyping success rate was 63.6% (18,343/28,820) for *Pfcrt* K76T and 93.5% (16,232/17,365) for *Pfmdr*-*1* 86Y mutations. Prevalence of *Pfcrt* 76T was as follows; East Africa 48.9% (2528/5242), Southern Africa 18.6% (373/2163), West Africa 58.3% (3321/6608), Asia 80.2% (1951/2436). Prevalence of *Pfmdr*-*1* 86Y was; East Africa 32.4% (1447/5722), Southern Africa 36.1% (544/1640), West Africa 52.2% (1986/4200), Asia 46.4% (1276/2217). Over half, 52.6% (20/38) of included studies reported continued unofficial chloroquine use following policy change. Studies done in Madagascar and Kenya reported re-emergence of chloroquine sensitive parasites (IC_50_ < 30.9 nM). The average time (years) since discontinuation of chloroquine use to data collection was 8.7 ± 7.4. There was high heterogeneity (I^2^ > 95%).

**Conclusion:**

The prevalence of chloroquine resistance alleles among *Plasmodium falciparum* parasites have steadily declined since discontinuation of chloroquine use. However, *Pfcrt* K76T and *Pfmdr*-*1* N86Y mutations still persist at moderate frequencies in most malaria affected countries.

## Background

Chloroquine was once an important medicine used in malaria treatment especially due to its affordability, ease of use and high anti-malarial efficacy. However, due to high level of resistance among *Plasmodium falciparum* parasites, chloroquine was withdrawn from malaria treatment in most malaria endemic countries [1]. It is estimated that the loss of chloroquine to resistance was responsible for more than doubling of malaria-associated mortality in sub-Saharan Africa, a region which bears over 90% of malaria burden [2, 3].

Chloroquine resistance reached fixation levels across malaria endemic countries by late 1990s [4]. As a result artemisinin agents and their derivatives were introduced in malaria treatment and have since been the first-line anti-malarial agents [3]. The use of artemisinin-based combination therapy (ACT) in malaria treatment, however, is limited by the high cost, pill burden and currently emerging risk of decreased *P. falciparum* parasite sensitivity [5–7]. Due to lack of current effective alternative agents to ACT, malaria treatment faces uncertain future which could expose populations most affected by the disease to the risk of increased malaria-associated mortality as previously seen with chloroquine [2].

Recent studies have indicated emergence of *P. falciparum* parasites susceptible to chloroquine following cessation of its use [4, 8]. However, considerations to re-introduce chloroquine in malaria treatment is faced with the challenge of inadequacy of information on current extent of chloroquine sensitivity and the uncertainty over how this might affect future resistance. The current review was intended to collate evidence and provide current evidence on genotypic and phenotypic chloroquine resistance among *P. falciparum* parasites in malaria affected countries.

## Methods

### Protocol development

A systematic review protocol was developed following STREGA [9] and PRISMA-P [10] guidelines. The protocol was registered in International Prospective Register of Systematic Reviews, PROSPERO (#CRD42018083957, http://www.crd.york.ac.uk/prospero) and published in a peer-reviewed journal [11].

### Review question

The review sought to establish the prevalence of *Pfcrt* K76T and *Pfmdr1* N86Y alleles among *Plasmodium falciparum* parasites in malaria affected countries since official discontinuation of chloroquine use in malaria treatment.

### Search strategy

#### Electronic search

Electronic search for Pubmed data base is reported in the published protocol [11]. The search terms were combined using Boolean logic ‘OR’ for synonymous terms and ‘AND’ across elements of PECOS (Population, Exposure, Comparison, Outcome and Study design).

The search terms used included, Chloroquine, ‘Antimalarial drug’, 4-aminoquinoline, ‘antimalarial agent’, Amodiaquine, piperaquine, ‘*Plasmodium falciparum*’, ‘malaria parasites’, ‘parasite sensitivity’, sensitivity’, ‘susceptibility’, ‘parasite susceptibility’, extent, spread, prevalence, occurrence, proportion, frequency, resistance, ‘resistance alleles’, ‘resistance mutations’, polymorphisms, ‘resistance reversal’, ‘*Pfcrt* K76T’, ‘*Pfmdr1* N86Y’, ‘resistance reversal’, mutations, ‘1990-to-February 2018’. The search terms were restricted to title and abstract during article search in each data base. There was no language restriction in article search, articles not published in English were translated using Google translator before screening for inclusion or exclusion.

#### Additional searches

The reference lists of included articles were screened and for any reference that could potentially be eligible for inclusion in the review, a full text article was retrieved. In addition, authors of included articles were contacted for any relevant publications on the review topic but did obtain any response.

#### Data management

**Fig. 1:**
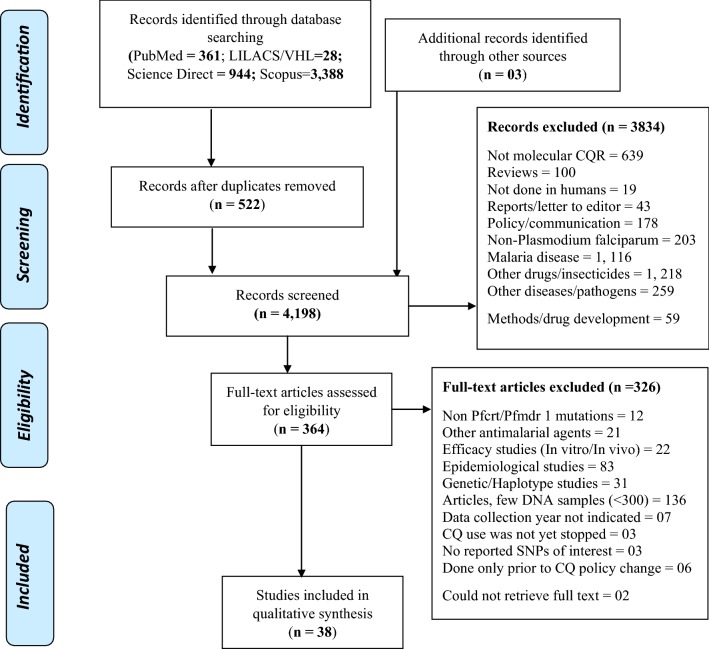
Prisma diagram showing flow of article search and screening

All article citations retrieved from database searches were exported into EndNote software version X7 (Thomson Reuters, 2015) and duplicates removed. The articles were grouped into relevant categories as indicated in the PRISMA flow diagram (Fig. 1).

### Selection of studies in the review

#### Eligibility criteria

The titles and abstracts were screened using a priori criteria [11]. Articles that reported on at least one of the chloroquine resistance alleles, *Pfcrt* K76T or *Pfmdr1*-N86Y were considered for inclusion. Articles that reported prevalence of the above chloroquine resistance alleles and genotyped more than 300 *P. falciparum* DNA samples were included. Studies that reported multiple parasite resistance genotype infections among patients were included in the review. Studies that assessed prevalence of resistance alleles (*Pfcrt* K76T or *Pfmdr1*-N86Y) following cessation of chloroquine use in malaria treatment were included. The review included studies that assessed prevalence of chloroquine resistance alleles both before and after official discontinuation of chloroquine use in malaria treatment [11].

#### Exclusion criteria for ineligible studies

For exclusion, the following articles were considered for exclusion; focused on other drugs/insecticides, other malaria parasites, reviews, non-molecular studies, non-human studies, method development, non-*Pfcrt/Pfmdr*-*1* genotypes, epidemiological studies, In vitro efficacy studies only without genotypic analysis, genetic studies, those that did not report any single nucleotide polymorphisms (SNPs). The articles were also considered for exclusion if they were letters to the editor/policy/communication, year of data collection is not indicated, chloroquine still being officially used after change in malaria treatment policy, data collected from countries that have not officially stopped the use of chloroquine in malaria treatment, and studies that screened few *P. falciparum* DNA (< 300 samples). Citations whose full text articles could not be retrieved (02) were considered for exclusion (Fig. 1).

#### Minimizing bias in article identification, selection and data extraction

A second librarian (RS), validated electronic search in PubMed by performing an independent and duplicate search. A second reviewer (EAO) screened all full text articles excluded by the first reviewer (MO). Any discrepancies among the reviewers were resolved by discussion and consensus. Two reviewers (NL and BB) performed duplicate and independent data extraction. Any disagreement between the reviewers was resolved by discussion and consensus. Any further disagreement between the two reviewers was referred to the third reviewer for a final decision (MO).

#### Data extraction

Data extraction form was developed in Excel spread sheet 2007, pre-tested on 5 articles and adjusted as appropriate. The following data was extracted from included articles, author, citation, country where study was done, study design, method of sample collection, years covered by data collection, year when chloroquine use was officially stopped, duration (years) since discontinuation of chloroquine use, whether chloroquine is still being used in the study area, In vitro assay (IC_50_), genotyping success rate, DNA extraction method, laboratory where genotyping was done, prevalence of *Pfcrt* 76T before and after cessation in chloroquine use, prevalence of *Pfmdr*-*1* 86Y before and after discontinuation of chloroquine use, trends in prevalence of chloroquine resistance alleles (*Pfcrt* 76T, *Pfmdr*-*1* 86Y), nature of malaria transmission, prevalence of mixed genotype infections, prevalence of other mutations (184F, 1246D, 1042D), and factors associated with chloroquine malaria treatment outcomes [11].

#### Data synthesis of included studies

Descriptive data synthesis was conducted with findings from a single primary study being the unit of analysis. Sub-groups were created, region (West Africa, East Africa, Southern Africa, and Asia), study-design (cross sectional, cohort, RCTs and non-randomized clinical trials). Extracted data was analyzed using Open Meta Analyst software [12]. Descriptive summaries of study outcomes were generated including; year of official cessation of chloroquine use, chloroquine in vitro IC_50_, duration from discontinuation of chloroquine use to data collection, chloroquine resistance alleles, factors associated with chloroquine resistance, genotyping success rate, allele calling algorithms, chloroquine use, and trends in genotypic chloroquine resistance.

DerSimonian–Laird (DL) random effects analysis was performed to establish a summary estimate of prevalence of *P. falciparum Pfcrt* 76T and *Pfmdr1* 86Y resistance alleles in malaria affected regions using Open Meta Analyst software. Sub-group analysis was performed, region (East Africa, Southern Africa, West Africa, Asia) and study design (cross sectional, Non-randomized clinical trials and RCTs). Heterogeneity in included articles was inferred from the summary estimates of I^2^- statistic. The I^2^ statistic was used to indicate percentage (%) heterogeneity that could be attributed to between-study variance. Interpretation: I^2^ = 25% (small heterogeneity), I^2^ = 50% (moderate heterogeneity), I^2^ = 75% (large heterogeneity) [13]. Due to high level of heterogeneity in the included studies the authors were unable to perform quantitative data analysis.

#### Missing data and risk of bias assessment

The variables that were missing from included articles were recorded as not reported. No statistical test was applied in handling missing data. However, available information was used in recalculating some variables in addition to contacting authors. The risk of publication bias was assessed using indirect assessment of rank correlation between effect size and sample size (Kendall’s tau) method [14]. In this method correlation of the articles is interpreted from the analysis output in Open Meta (Analyst) software where 1, represent perfect correlation and 0 no correlation.

## Results

### Description of included studies

Article search yielded a total of 4721 citations (Pubmed = 361, Lilacs/Vhl = 28, Science Direct = 944, Scopus = 3388). Additional searches resulted in 03 articles. After removing duplicates, 4198 articles remained and their titles and abstracts were screened for inclusion using a priori criteria. A total of 3, 834 articles were excluded after title and abstract screening. Full text of the remaining 364 articles were obtained and screened using a set criteria. Two (02) citations were excluded as their full text could not be obtained. A total of 326 articles were excluded as they did not meet the a priori inclusion criteria. Review data was extracted from a total of 38 articles that met the a priori inclusion criteria (Fig. 1).

### Characteristics of included studies

Eleven (11) studies were conducted in East Africa, six (6) were from Southern Africa, fourteen (14) from West Africa and seven (7) from Asia (Table 1). A total of twenty-six (26) studies were cross-sectional, eight (08) RCTs, three (03) non-randomized clinical trials and one (01) cohort study.

**Table 1:** Summary of the included articles

Author	Year	Country	Design	Study period	Year CQ PC	Years CQ not in use	CQ still used	Prev. of *Pfcrt* K76T after CQ policy change	Prev. of *Pfmdr1* N86Y after CQ policy change
Andriantsoanirina [37]	2010	Madagascar	RCT	2006–2007	2005	01	Yes	0.32% (2/621)	44.5% (147/330)
Achieng [19]	2015	Kenya	CSS	1995–2014	BF/AF	NA	Yes	28.5% (212/745)	14.9% (111/745)
Asih [45]	2009	Indonesia	CSS	2007	2004	3	NR	89.2% (345/387)	42.3% (82/194)
Atroosh [36]	2012	Malaysia	CSS	2007–2011	NR	NA	Yes	52.7% (39/75)	5.3% (4/75)
Baraka [15]	2015	Burkina Faso	RCT	NR	2005	NA	NR	48.7% (130/272)	61.8% (168/267)
Baraka [76]	2018	DRC/Uganda	RCT	2012–2014	NR	NR	NR	NR	DRC: 37.9% (278/732) Uganda: 7.5% (55/732)
Bo Huang [35]	2016	Grande Comore Island	CSS	2006–2014	2004	2	Yes	49.5% (100/202)	66.8% (135/202)
Sutar [41]	2013	India	CSS	2008	1982	26	No	77.6% (156/201)	59.5% (119/200)
Das [28]	2017	India	CT	2008–2013	1982	26	Yes	Kolkata: 69.1% (540/781) Purulia: 50.97% (398/781)	Kolkata: 44.3% (346/781) Purulia: 65.6% (512/781)
Frank [29]	2011	Gabon	CSS	1995–2007	2003	8	Yes	99.2% (356/359)	NR
Gadalla [77]	2015	Tanzania	Cohort	2003–2006	2001	2	NR	93.6% (219/234)	91.6% (163/234)
Gupta [42]	2018	Mozambique	CSS	2015	2002	13	NR	2.3% (8/351)	3.1% (11/351)
Hemming-Schroeder [20]	2018	Kenya	CSS	2003–2015	1999	4	Yes	54.5% (335/615)	24.2% (142/586)
Akala [46]	2014	Kenya	CSS	2008–2012	1999	9	NR	42.2% (326/772)	20.5% (165/804)
Jovel [34]	2014	Guinea-Bissau	CT	2001–2002	2008	7	Yes	36.1% (600/1662)	36.7% (606/1650)
Kateera [40]	2016	Rwanda	CSS	2015	2001	14	No	39.2% (152/388)	3.7% (14/382)
Asare [26]	2014	Ghana	CSS	2012	2004	8	Yes	72% (154/214)	NR
Lekana-Douki [39]	2011	Gabon	CSS	2004/2009	2005	4	No	95.2% (219/230)	66.5% (153/230)
Lucchi [22]	2015	Kenya	CSS	2010–2013	1999	11	Yes	9.9% (20/203)	3.5% (7/200)
Ly [30]	2012	Senegal	CSS	2000–2009	2003	BF/AF	Yes	59.2% (316/532)	59.2% (316/532)
Afsharpad [44]	2012	Iran	CSS	2008–2010	2007	1	NR	94.7% (161/170)	42.9% (73/170)
Mbogo [24]	2014	Uganda	CT	2003–2012	2004	1	Yes	94.3% (807/856)	34.6% (267/771)
Mungthin [33]	2014	S. Thailand	CSS	2009	1995	14	Yes	100% (558/558)	89.2% (498/558)
Mittra [43]	2006	India	CSS	2000–2004	1982	26	NR	84.2% (223/265)	30.1% (72/239)
Mwai [21]	2009	Kenya	CSS	1993–2006	1999	NA	Yes	63% (203/322)	74.6% (126/169)
Mwanza [38]	2016	Zambia	CSS	2010–2013	2003	7	No	0	NR
Ndam [32]	2017	Cameroon	CT	2003–2012	2002	1	Yes	42.9% (111/259)	NR
Ndiaye [31]	2012	Senegal	CSS	2009–2011	2003	6	Yes	18.7% (84/449)	NR
Ogouyemi-Hounto [23]	2013	Benin	CSS	2011	2004	7	Yes	93.9% (200/213)	57.1% (121/212)
Okombo [18]	2014	Kenya	CSS	1995–2013	2004	9	Yes	57.9% (212/366)	51.6% (119/231)
Afoakwah [27]	2014	Ghana	CSS	2010–2011	2004	6	Yes	58.5% (144/246)	NR
Otienoburu [47]	2016	Liberia	CT	2008–2009	2003	5	NR	93.5% (275/294)	69.4% (204/294)
Some [78]	2016	Burkina Faso	CT	2012	2005	7	NR	24.6% (58/236)	19.3% (46/238)
Sondo [79]	2015	Burkina Faso	CT	2010–2012	2005	5	NR	20.5% (120/584)	NR
Thomsen [16]	2013	Mozambique	CSS	2009–2010	2002	7	Yes	43.1% (163/378)	35.4% (134/378)
Raman [17]	2011	Mozambique	CSS	2006–2010	2002	4	Yes	76.9% (1694/2203)	0
Duah [25]	2013	Ghana	CSS	2003–2010	2005	BF/AF	Yes	52.1% (554/1063)	47.8% (372/778)
Mohammed [80]	2013	Tanzania	CSS	2010–2011	2001	9	NR	5.7% (42/741)	NR

*CSS* cross-sectional, *BF/AF* before/after, *NR* not reported, *CQ* chloroquine, *PC* policy change

### Genotyping errors and parasite DNA extraction methods

For *Pfcrt* 76T allele, genotyping was done in a total of 28,820 DNA samples with 18,343 being successfully genotyped, 63.6% (18,343/28,820). While genotyping was done for *Pfmdr*-*1* 86Y mutation in a total of 17,365 DNA samples with 16,232 being successfully genotyped, 93.5% (16,232/17,365). One study by Baraka et al. [15] done in Bukina-Faso did not report DNA extraction method used. The other 37 articles reported using either of the following DNA extraction methods, Chelex-100, Quiagen blood mini kit, Takara DNA blood mini kit, phenol–chloroform, Accu-Prep Genomic DNA extraction kit, methanol fixation method, QIAxtractor system, and Nucleospin Genomic DNA blood pure kit.

### Heterogeneity of included studies

Sub-group analysis was done based on regions from where the studies were done, East Africa, Southern Africa, West Africa and Asia. A high heterogeneity, I^2^ > 97% was observed in all regions where the studies were conducted (East Africa: *Pfcrt* 76T, I^2^ = 99.8%; *Pfmdr1* 86Y, I^2^ = 99.6%; Southern Africa: *Pfcrt* 76T, 99.2%; *Pfmdr1* 86Y, I^2^ = 99.4%; West Africa: *Pfcrt* 76T, I^2^ = 99.8%; *Pfmdr1* 86Y, I^2^ = 98.1%; Asia: *Pfcrt* 76T, I^2^ = 99.2%; *Pfmdr1* 86Y, I^2^ = 99.5%). Heterogeneity was further assessed in sub-groups based on study designs (Cross-sectional, RCTs and non-randomized trials). Heterogeneity was high in articles from all study designs, I^2^ > 95% (RCTs: *Pfcrt* 76T, I^2^ = 99.7%; *Pfmdr1* 86Y, I^2^ = 99.3%; Non-randomized trials: *Pfcrt* 76T, I^2^ = 99.4%; *Pfmdr1* 86Y, I^2^ = 99.2%; Cross-sectional studies: *Pfcrt* 76T, I^2^ = 99.9%; *Pfmdr1* 86Y, I^2^ = 99.6%).

### Aggregate prevalence of chloroquine resistance alleles in malaria affected regions

In East Africa, average prevalence of *Pfcrt* K76T resistance alleles is 48.9% (2528/5242) (95% CI: 22.5–75.2%) and *Pfmdr*-*1* 86Y is 32.4% (1447/5722) (95% CI 19.2–45.5%). In Southern Africa, average prevalence of *Pfcrt* K76T resistance allele is 18.6% (373/2163) (95% CI 14–23.1%), and *Pfmdr1* 86Y is 36.1% (544/1640) (95% CI 12.7–59.4%). In West Africa, average prevalence of *Pfcrt* K76T resistance alleles is 58.3% (3321/6608) (95% CI 40.4–76.2%) and *Pfmdr1*-86Y is 52.2% (1986/4200) (95% CI 41.1–63.3%). In Asia, the average prevalence of *Pfcrt* 76T resistance allele is 80.2% (1951/2436) (95% CI 68–92.4%) and *Pfmdr1* 86Y is 46.4% (1276/2217) (95% CI 22.3–70.4%).

A total of 26 studies were conducted following cross-sectional study design and reported average prevalence of chloroquine resistance alleles as *Pfcrt* 76T, 54.3% (5179/10,333) (95% CI 31.7–77%), *Pfmdr*-*1* 86Y, 37.7% (2765/7446) (95% CI 25–50.4%). Three studies were done following non-randomized clinical trial design and reported average prevalence of chloroquine resistance alleles as *Pfcrt* 76T, 82.7% (1551/1931) (95% CI 64–101.3%), *Pfmdr1*-86Y, 50.6% (899/2038) (95% CI 26.5–76.4%). Eight studies used RCT design and reported average prevalence of chloroquine resistance alleles was *Pfcrt* 76T, 33.7% (1224/3951) (95% CI 14.4–52.9%), *Pfmdr1*-86Y, 36.6% (1258/3851) (95% CI 20–53.3%).

### Enforcement of policy on discontinuation of chloroquine use in malaria treatment

Over half, 57.9% (22/38) of included studies reported continued use of chloroquine after official discontinuation following change in malaria treatment policy. These included, Mozambique [16, 17]; Kenya [18–22]; Benin [23]; Uganda [24]; Ghana [25–27]; India [28]; Gabon [29]; Senegal [30, 31]; Cameroon [32]; Southern Thailand [33]; Guinea-Bissau [34]; Grande Comoros [35]; Malaysia [36] and Madagascar [37].

Of the 38 studies, only 4 (10.5%) done in Zambia [38], Gabon [39], Rwanda [40] and India [41] reported elimination of chloroquine use following official discontinuation (Table 1).

### Prevalence of chloroquine resistance alleles after discontinuation of chloroquine use in malaria treatment

#### *Pfcrt* K76T

Nine of the 38 studies were done after more than 10 years since cessation of chloroquine use. Of these studies, 4 reported less than 50% *Pfcrt* K76T allele frequency, Kenya (28.4%, [19]), Mozambique (2.3%, [42]), Rwanda (39.2%, [40]), and Kenya (9.9%), [22]). Two studies reported *Pfrct* 76T allele frequency of more than 80%, [33], 100% (Southern Thailand) and [43], 84.2% (India) (Table 1).

#### *Pfmdr1* N86Y

Four of the nine studies done after more than 10 years since change of chloroquine use reported less than 10% prevalence of *Pfmdr1* 86Y alleles, [22] (3.5%), [40] (3.7%), and [42] (2.3%). A study by Mungthin et al. [33] reported *Pfmdr1* 86Y allele prevalence of 89.1% 10 years after cessation of chloroquine use in malaria treatment (Table 1).

### Change in malaria treatment policy from chloroquine to sulfadoxine–pyrimethamine

Majority of countries officially stopped chloroquine use in malaria treatment in 2004 (Range 1982–2008). India was the first country to stop chloroquine use in 1982. Guinea-Bissau in West Africa continued using chloroquine in malaria treatment until 2008. The average duration of time since official change in malaria treatment policy to data collection of the individual included studies is 8.7 ± 7.4 (95% CI 6.1–11.3) (Table 1).

Five studies, 13.2% (5/38) done in Zambia, Gabon, Rwanda, and India reported elimination of chloroquine use after official discontinuation. In Zambia [38], there was no detectable chloroquine resistance alleles 7 years after cessation of chloroquine use. In Gabon, there was no detectable chloroquine use 1 year after official discontinuation of chloroquine use and prevalence of *Pfcrt* 76T was 95.2% and *Pfmdr*-*1* 86Y, 66.5% [39]. Fourteen years after change in malaria treatment policy in Rwanda chloroquine use was stopped and prevalence of resistance alleles was, *Pfcrt* 76T, 39.2% and *Pfmdr*-*1* 86Y, 3.7% [40]. In India, there was no chloroquine use for over two decades after change in policy and resistance allele frequency was *Pfcrt* 76T, 77.6% and *Pfmdr*-*1* 86Y, 59.5% [28, 41] (Table 1).

Multiple chloroquine resistance surveys were done in eight countries (Kenya, Tanzania, Mozambique, Burkina Faso, Ghana, India, Gabon and Senegal). In all the eight countries chloroquine resistance allele prevalence decreased with increasing duration of time since discontinuation of chloroquine use in malaria treatment. None of the eight countries recorded zero prevalence of *Pfcrt* 76T allele after discontinuation of chloroquine use. Only Mozambique reported zero prevalence of *Pfmdr1*-86Y allele [17] 4 years after cessation of chloroquine use. The lowest reported *Pfcrt* 76T resistance allele prevalence, 2.3% was observed in Mozambique [42] 13 years after discontinuation of chloroquine use in malaria treatment. In India, a study by Mittra [43] reported the highest prevalence, 84.2% of *Pfcrt* 76T resistance allele after over a decade after discontinuation of chloroquine use in malaria treatment.

The average *Pfcrt* K76T allele frequencies varied in different countries since cessation in chloroquine use, Kenya (42.7%), Tanzania (49.7%), Burkina Faso (31.3%), Ghana (60.2%), India (70.5%), Gabon (97.2%) and Senegal (38.9%).

The average prevalence of *Pfmdr*-*1* 86Y allele varied in different countries since change in malaria treatment policy from chloroquine to sulfadoxine-pyrimethamine, Kenya (31.6%), Mozambique (19.3%), Burkina Faso (40.6%), Ghana (47.8%), India (49.9%) and Senegal (59.2%).

### Prevalence of chloroquine resistance alleles before discontinuation of chloroquine use in malaria treatment

#### *Pfcrt* K76T

In Ghana, 2 years before change (2003–2004) in policy, *Pfcrt* 76T allele frequency was 50-98% [25]. In Kenya, *Pfcrt* 76T allele frequency varied from several reports prior to cessation of chloroquine use in malaria treatment, 57% (1995), 88.9% (1999) [18]; in 1993–2003, 62.8% [19]. In Senegal, [30] (72.4%), [31] (2001: 64%; 2004: 59.5%). In Gabon *Pfcrt* 76T allele frequency varied, 93.8% in 2004 [39], 100% in 1995–2002 [29]. In India prior to change in chloroquine use, *Pfcrt* 76T prevalence varied in different studies, 91% [41], 53% [28]. In Guinea-Bissau, *Pfcrt* 76T allele frequency before change in policy was, 23.6%. In Iran the reported frequency was 97.7% [44]. In Uganda, there was high reported frequency prior to change in policy, 99% [24]. In Zambia, *Pfcrt* 76T allele frequency was 95% in 2001 prior to policy change. A study by Bo Huang et al. 2016 reported 62-98% *Pfcrt* 76T allele frequency prior to policy change in Grande Comore Island. In Mozambique reported allele frequency prior to policy change was 96.1% [17] (Table 2).

**Table 2:** Prevalence of chloroquine resistance alleles before and after change in policy

Author	Year of CQ PC	Prev. of *Pfcrt* 76T BF CQ PC	Prev. of *Pfmdr*-*1* 86Y BF CQ PC	Prev. of *Pfrct* 76K/T AF CQ PC	Prev. of *Pfmdr*-*1* 86N/Y AF CQ PC	Prev. of 76T/86Y AF CQ PC	Prev. of Y184F AF CQ PC	Prev. of D1246Y AF CQ PC
Andriantsoanirina [37]	2005	NR	NR	NR	3.3%	NR	71.7%	33.3%
Achieng et al. [19]	BF/AF	62.8% (1993–2003)	NR	9.8%	11.4%	NR	40.8%	13.8%
Asih et al. [45]	2004	NR	NR	NR	NR	18.7% (65/348)	NR	63.3%
Atroosh et al. [36]	NR	NR	NR	NR	NR	NR	NR	4%
Baraka et al. [15]	2005	NR	NR	21.7%	19.5%	NR	NR	NR
Baraka et al. [76]	NR	NR	NR	NR	DRC: 9.3% Uganda: 1.5%	NR	DRC: 43.5% Uganda: 32.8%	DRC: 9.2% Uganda: 20.9%
Bo Huang et al. [35]	2004	62–98%	90–100%	NR	NR	NR	42.6%	20.8%
Sutar et al. [41]	1982	91% (2005)	91% (2005)	6.97%	9.5%	70% (109/156)	NR	53.19% (2013)
Das et al. [28]	1982	53% (2005)	NR	NR	16.13%	NR	NR	NR
Frank et al. [29].	2003	100% (’95–’02)	NR	NR	NR	NR	NR	NR
Gadalla et al. [77]	2001	NR	NR	1.7%	8.99%	NR	2.2%	82%
Gupta et al. [42]	2002	NR	NR	NR	NR	NR	46.7%	NR
Hemming-Schroeder et al. [20]	1999	NR	NR	12.7% (46/362)	23.7%	NR	33.3%	25.4%
Akala et al. [46]	1999	NR	NR	33.7% (260/772)	17.7%	NR	NR	NR
Jovel et al. [34]	2008	23.6%	43.1%	NR	NR	13% (80/615)	42%	NR
Kateera et al. [40]	2001	NR	NR	10.1% (39/388)	15.4%	NR	59.8%	19.2%
Lekana-Douki et al. [39]	2005	93.8% (2004)	75% (2004)	1.5% (2/134)	8.2%	NR	NR	5.2%
Lucchi et al. [22]	1999	NR	NR	13.7%	3%	NR	37.3%	6.8%
Afsharpad et al. [44]	2007	97.7%	41%	NR	2.4%	NR	NR	NR
Mbogo et al. [24]	2004	99% (2003–2004)	19% (2003–2004)	4% (31/776)	58.1%	NR	12.2%	40.4%
Mungthin et al. [33]	1995	NR	NR	NR	NR	NR	10.4%	NR
Mittra et al. [43]	1982	NR	NR	4.91%	69.1%	NR	99.16%	NR
Ndam et al. [32]	2002	NR	NR	19.7% (51/259)	NR	NR	NR	NR
Ndiaye et al. [31]	2003	65% (2000) 64% (2001) 59.5% (‘04)	NR	10.2% (46/449)	NR	NR	NR	NR
Ogouyemi-Hounto [23]	2004	NR	NR	NR	28.8%	55.2%	NR	NR
Okombo et al. [18]	2004	1995: 57% 1999: 88.9%	1995: 57.1% 1999: 72.8%	4.2% (8/192)	NR	NR	30.9%	19.1%
Otienoburu et al. [47].	2003	NR	NR	2.4% (7/294)	16%	NR	35.4%	18.7%
Some et al. [78]	2005	NR	NR	NR	NR	NR	70.8%	NR
Thomsen et al. [16]	2002	90%	NR	NR	NR	35.8% (59/165)	28.8%	NR
Raman et al. [17]	2002	96.1%	NR	NR	16%	NR	NR	NR
Duah et al. [25].	2005	2003: 50–98%	2003: 48–98%	NR	NR	NR	2010: 40–80%	2010: 35%
Sondo et al. [79]	2005	NR	NR	20.5% (120/584)	NR	NR	NR	NR

*NR* not reported, *PC* policy change, *CQ* chloroquine, *BF* before, *AF* after

#### *Pfmdr1* N86Y

In Ghana from 2003-to-2004 prior to policy change, *Pfmdr1* 86Y allele frequency was 48-96%. In Kenya a study by Okombo et al. [18] reported 57.1% in 1995 and 72.8% in 1999. In 2003-2004 a study by Mbogo et al. [24] in Uganda reported *Pfmdr1* 86Y allele frequency of 19%. A study by Afsharp et al. [44] in Iran reported allele frequency of 41% prior to policy change. In India, a study by Sutar et al. [41] reported 91% allele frequency. A study by Bo Huang et al. [35] in Grande Comore Island reported 90–100% prevalence. In Guinea-Bissau, *Pfmdr1* 86Y allele frequency prior to change in policy was 43.1% (Table 2).

Majority, 63.2% (24/38) of the studies did not report the prevalence of *P. falciparum* chloroquine resistance alleles prior to cessation of chloroquine use in malaria treatment (Table 2). A high proportion, 65.8% (25/38) of the studies reported occurrence of mixed genotype infections. Six studies reported mixed 76T/86Y infections (Indonesia: [45]; India: [41]; Kenya: [46]; Benin: [23]; Mozambique: [16]). Thirteen studies reported presence of 76T/K and 86Y/N genotype in *P. falciparum* infections. Two studies [28, 41] in India reported the presence of all three genotypes 76T/K, 86Y/N and 76T/86Y in a single parasite infection (Table 2).

### Phenotypic chloroquine resistance among *P. falciparum* after cessation of chloroquine use

Five studies reported phenotypic parasite resistance to chloroquine after official discontinuation of use in malaria treatment. In Madagascar, one (01) year after change of policy, chloroquine IC_50_ was 18.7 nM (95% CI 14.7–23.7 nM) [37]. India that stopped chloroquine use over two decades (26 years) prior to the study, chloroquine IC_50_ were, 2008 (Kolkata: 146.5 nM; Purulia: 162.25 nM), 2013 (Kolkata: 238.6 nM; Purulia: 247.42 nM) [28]. After over one decade since change of malaria treatment policy in Kenya, chloroquine IC_50_ values varied from year to year, 2010 (31.77 nM), 2011 (23.42 nM), 2012 (21.09 nM) and 2013 (19.85 nM) [22]. A study in Southern Thailand [33], 14 years after change in policy reported high average chloroquine IC_50_ value, 129.2 ± 45.2 nM. A study by Mwai et al. [21] in Kenya showed that *P. falciparum* parasites carrying *Pfcrt* 76T mutations had chloroquine IC_50_ of 63 ± 90 nM while those carrying *Pfmdr1* 86Y mutation had IC_50_ of 68 ± 87 nM (Table 3).

**Table 3:** Trends in parasite resistance after cessation of chloroquine use in malaria

Author	Mean IC_50_ in parasites with *Pfct* K76T	Country where study was done	Year policy was changed	Trends in prev. of 76T CQR allele AF PC	Trends in prev. of 86Y CQR allele AF PC
Andriantsoanirina et al. [37]	18.7 nM (95% CI 14.7–23.7 nM)	Madagascar	2005	NR	NR
Achieng et al. [19]	NR	Kenya	1999	2008–2014: 28.5%, 2014: 2.3%	2008–2014: 14.9%
Hemming-Schroeder et al. [20]	NR	Kenya	1999	Kakamega 2003: 80% 2005: 61% 2008: 60% 2015: 2.7% Kombewa 2003: 71% 2005: 91.9% 2008: 90% 2015: 11.8%	Kakamega 2003: 59.2% 2005: 59.1% 2008: 40% 2015: 4.2% Kombewa 2003: 57.1% 2005: 40% 2008: 45% 2015: 5%
Akala et al. [46]	NR	Kenya	1999	2008: 68.4% 2009: 55.4% 2010: 47.8% 2011: 12.4% 2012: 29.8%	2008: 38.1% 2009: 24.3% 2010: 19.7% 2011: 7.8% 2012: 13.3%
Lucchi et al. [22]	2010: 31.77 nM 2011: 23.42 nM 2012: 21.09 nM 2013: 19.85 nM	Kenya	1999	2010: 38.8% 2011: 28.6% 2012: 18.7% 2013: 7%	2010: 2% 2011: 1.5% 2012: 0% 2013: 0%
Okombo et al. [18]	NR	Kenya	1999	2006: 49.5% 2013: 17.2%	2006: 57.5% 2013: 2.1%
Mwai et al. [21]	63 ± 90 nM (5-150 nM)	Kenya	1999	1993–2006: 94% to 63%	1993–2006: 75%
Bo Huang et al. [35]	NR	Grande Comore Island	2004	2006–2014: 72.2–19.5%	2006–2007: 87% 2013–2014: 40.2%
Das et al. [28]	Kolkata 2013: 238.6 nM, (95% CI 121–321 nM) Purulia 2013: 247.42 nM, (95% CI 126–316 nM)	India	1982	Kolkata 2012: 94.64% Purulia 2012: 96.15%	Kolkata 2013: 87.23% 2009: 46.67% Purulia 2013: 87.93% 2009: 64.2%
Frank et al. [29]	NR	Gabon	2003	1995–2002: 100%; 2005–2007: 97%	NR
Gadalla et al. [77]	NR	Tanzania	2001	2003: 96.9% 2004: 94.9% 2005: 89.5% 2006: 100%	2003: 67.7% 2004: 67.8% 2005: 66.2% 2006: 75%
Jovel et al. [34]	NR	Guinea-Bissau	2008	2008: 31% 2010: 48% 2011: 57% 2012: 34%	2008: 36% 2010: 30% 2011: 29% 2012: 34%
Ly [30]	NR	Senegal	2003	2004–2005: 47.16%, 2006–2009: 59.46%	
Afsharpad et al. [44]	NR	Iran	2007	2008–2010: 94.7%	2008–2010: 42.9%
Mbogo et al. [24]	NR	Uganda	2004	2005: 100% 2007: 100% 2008: 98% 2009: 94% 2010: 96% 2011: 92% 2012: 67%	2005 (53%) 2007 (49%) 2008 (16%) 2009 (44%) 2010 (19%) 2011 (16%) 2012 (8%)
Mwanza et al. [38]	NR	Zambia	2003	2001: 95% 2006: 26% 2010–2013: 0%	NR
Ndam et al. [32]	NR	Cameroon	2002	2003: 53% 2012: 25.3%	NR
Ndiaye et al. [31]	NR	Senegal	2003	2009: 19.8% 2010: 18.2% 2011: 18%	NR
Sondo et al. [79]	NR	Burkina Faso	2005	2010: 27.22% 2012: 16.49%	NR
Thomsen et al. [16]	NR	Mozambique	2002	2009: 55.9% 2010: 33.8%	2009: 47.2% 2010: 26.9%
Raman et al. [17]	NR	Mozambique	2002	2006: 96.1% 2007: 91.3% 2008: 76.1% 2009: 45.5% 2010: 32.4%	2006: 74.7% 2007: 76.4% 2008: 64.9% 2009: 51.6% 2010: 30.9%
Duah et al. [25]	NR	Ghana	2005	2005–2006:73–95% 2007–2008:50–95%, 2010: 45–80%	2005–2006:31–67% 2007–2008:36–67% 2010: 10–50%

*CQR* chloroquine resistance, *PC* policy change, *BF* before, *AF*: after, *NR* Not reported, *Prev.* prevalence

### Factors associated with chloroquine treatment outcomes in malaria patients

A study in Madagascar [37] showed that patients with *P. falciparum* parasites having a *Pfmdr*-1 86Y mutation (OR = 4.6, 95% CI 2.3 to 8.9), and age (OR = 1.2, 95% CI 1.1 to 1.3) were predictors of chloroquine treatment failure among malaria patients. Patients with *P. falciparum* parasites having *Pfcrt* 76T are more likely to fail on treatment Malaysia, [36]. A study in Ghana [26] showed that being infected with parasites carrying *Pfcrt* 76T mutation was associated with staying in a place where CQ is sold and used for malaria treatment (P < 0.0001). In India (Purulia), chloroquine treatment failure was strongly associated with *P. falciparum* parasites carrying *Pfmdr1* 86Y+ 1246Y mutation in 2008–2009. Lucchi et al. indicated that *P. falciparum* parasites with *Pfcrt* 76T and *Pfmdr*-*1* 86Y mutation had significantly higher IC_50_ values compared to wild-type in Kenya [22], and in Senegal [30]. A study by Afoakwah et al. [27] (Iran) showed that patients infected with *P. falciparum* parasites having *Pfcrt* 76T mutation had higher parasite density with mean density of 73,529 parasites/µl of blood compared to wild type infections.

### Duration since official cessation of chloroquine use and prevalence of resistance alleles

Thirty-two (32) of the 38 included studies (84.2%) reported time duration (years) since discontinuation of chloroquine use to when data collection was done. Of which the average time (years) since cessation of chloroquine use in malaria treatment up to data collection date was 8.7 ± 7.4 (95% CI 6.1–11.3) years. India stopped use of chloroquine in malaria treatment for more than two decades (26 years) prior to data collection in all included studies.

Fifteen studies were conducted in regions/countries less than 5 years after discontinuation of chloroquine use in malaria treatment and reported average prevalence, 63.5% of *Pfcrt* 76T resistance allele. Twelve studies done in the same regions and time-period reported average prevalence, 52.2% of *Pfmdr1* 86Y. Twelve studies were conducted in countries between 6 and 10 years after cessation of chloroquine use and reported average prevalence, 49.1% of *Pfcrt* 76T resistance alleles. Seven studies conducted in the same countries and time-period reported average prevalence, 37.4% of *Pfmdr1*-86Y. Eight studies were conducted in countries after more than 10 years since cessation in use of chloroquine in malaria treatment and reported average prevalence of resistance alleles, *Pfcrt* 76T (56.5%) and *Pfmdr1* 86Y (38.8%).

A study by Mungthin et al. [33] conducted in Southern Thailand 14 years since cessation of chloroquine use reported 100% prevalence of *Pfcrt* 76T resistance alleles. In Gabon, Frank et al. [29] reported high prevalence, 99.2% of *Pfcrt* 76T resistance alleles 8 years after change in chloroquine policy in malaria treatment. In Benin, Ogouyemi-Hounto et al. [23] reported 93.9% prevalence of *Pfcrt* 76T 7 years after cessation of chloroquine use. In Liberia, 93.5% prevalence of *Pfcrt* 76T resistance alleles was reported in a study done 5 years after cessation of chloroquine use in malaria treatment [47].

The prevalence of chloroquine resistance alleles varied in different malaria endemic countries at the time of discontinuation of chloroquine use in malaria treatment. Some countries had over 90% prevalence of *Pfcrt* 76T resistance allele, 94.3% (Uganda: [24]), 94.7% (Iran: [44]), 95.2% (Gabon: [29]). Madagascar had the lowest prevalence, 0.32% of *Pfcrt* 76T 1 year after change in treatment policy [37].

## Discussion

Chloroquine resistance was first detected in Thailand in Southeast Asia [48] and spread to other malaria affected regions [49, 50]. Due to high levels of resistance, chloroquine was withdrawn from malaria treatment globally except in Central America where parasites remain susceptible [1]. The review observed progressive decline in prevalence of *Pfcrt* 76T and *Pfmdr*-1 86Y resistance alleles following official discontinuation of chloroquine use across malaria endemic countries. This finding is similar to previous review which indicated a reduction in prevalence of chloroquine resistance alleles, *Pfcrt* 76T and *Pfmdr*-*1* 86Y since discontinuation of chloroquine use [51]. The current review further highlights presence of phenotypic *P. falciparum* chloroquine sensitivity. In Madagascar, a study by Andriantsoanirina et al. [37] reported chloroquine IC_50_, 18.7 nM (sensitive), India, [28], 146.5 nM, 162.25 nM (highly resistant), Kenya, < 25 nM (sensitive), Southern Thailand, [33], 129.2 nM (highly resistant) (highly resistant (IC_50_ > 101 nM), moderately resistant (IC_50_ 30.9 nM < IC_50_ < 100.9 nM) and sensitive (IC_50_ < 30.9 nM) [52]. The observed re-emergence of chloroquine susceptibility is timely especially due to recent emergence of *P. falciparum* parasites with decreased artemisinin susceptibility in Southeast Asia [6, 7]. Chloroquine sensitive *P. falciparum* parasites that have recently emerged especially in Africa following discontinuation of chloroquine use have evolved independently from African parasites [53]. Immune individuals in high malaria transmission settings serve as reservoirs [54] and are likely to be the source of current observed chloroquine susceptible parasite strains that have emerged following cessation of chloroquine use [53].

A previous review by Venkatesan et al. [51] showed continued prevalence of *Pfcrt* 76T alleles in malaria endemic regions, East Africa (67.6%), and West Africa (73.3%) following discontinuation of chloroquine use. The current review observed a further decline in *Pfcrt* 76T prevalence rates from those reported by Venkatesan et al. [51], East Africa (48.9%, 2528/5242), Southern Africa (18.6%, 373/2163), West Africa (58.3%, 3321/6608) and Asia (80.2%, 1951/2436). A similar trend in *Pfmdr1* 86Y mutation was seen by Ventaketsan et al. [51] in different regions of Africa, East Africa (61.1%), and West Africa (48.7%). A recent review by Okell et al. [55] also indicated a reduction in prevalence of *Pfmdr1* 86Y gene following change in malaria treatment policy. The current review observed lower average prevalence rate of *Pfmdr*-*1* 86Y mutation, East Africa (32.4%, 1447/5722), Southern Africa (12.7%, 544/1640), West Africa (52.2%, 1986/4200), and Asia (22.3%, 1276/2217). Despite an overall decline in frequency, chloroquine resistance alleles have persisted in malaria endemic countries following official change in policy more than a decade ago. Persistence of chloroquine resistance alleles in the population could be due to development of compensatory mutations in *P. falciparum* parasites that restore parasite fitness in the absence of drug pressure [56]. This incomplete reversal of resistance is a drawback to efforts considering potential re-emerging role of chloroquine in malaria control and elimination efforts. Chloroquine resistance alleles *Pfcrt* 76T and *Pfmdr*-*1* 86Y are selected for by the current artemisinin-based combinations used in malaria treatment [57, 58]. The wide spread use of artemisinin agents in malaria treatment across malaria endemic regions could be contributing to the observed persistence of chloroquine resistance alleles in the population. Duraisingh et al. [59] indicated that increased prevalence of *Pfmdr*-*1* 86 N alleles was associated with decreased *P. falciparum* parasite sensitivity to lumefantrine and mefloquine. The persistence of *P. falciparum* parasites carrying *Pfcrt* 76T and *Pfmdr*-*1* 86Y mutations could indicate the high efficacy of artemisinin and partner drug (lumefantrine) especially among African parasites [51, 60].

The review observed varying extents of decline in prevalence of *Pfcrt* 76T and *Pfmdr*-*1* 86Y chloroquine resistance alleles across malaria affected countries following discontinuation of chloroquine use. In addition, chloroquine resistance allele frequencies prior to policy change varied across malaria endemic countries. The drivers for observed variations in rates of re-emergence of chloroquine sensitive *P. falciparum* genotype are not clear. However, continued chloroquine use after official discontinuation, parasite biology, fixation of chloroquine resistance alleles prior to change in malaria policy could be having a role. A study by Laufer et al. [53] indicated that the re-emerging susceptible *P. falciparum* parasite strains are an expansion of susceptible parasite population that survived during the period when chloroquine was still being used for malaria treatment. Therefore, the observed variation in prevalence of chloroquine resistance alleles could also be due to differences in baseline levels of *Pfcrt* 76T and *Pfmdr*-*1* 86Y alleles in the population at the time of discontinuation of chloroquine use across malaria endemic regions. Drug-susceptible organisms may regain predominance as long as there is a population that survives due to exposure to non-lethal drug concentration despite prolonged drug pressure. The review showed that the rate of decline of prevalence of *Pfcrt* 76T and *Pfmdr*-*1* 86Y resistance alleles do not correspond to the duration of time since cessation in chloroquine use in most malaria endemic countries. Previous studies have shown that the extent of resistance corresponds to the prevailing drug pressure [61].

Continued use of chloroquine was observed in majority of malaria affected regions. This may be indicative of the challenges in implementation of malaria treatment policies across malaria affected countries [5]. This is especially the case in sub-Saharan Africa where chloroquine remained accessible to the general population through unofficial channels. In some countries, chloroquine continued to be imported for use in other indications such as *P. vivax* infections, and inflammatory conditions such as arthritis [5]. The ease of access of medicines over the counter in most low and middle income countries provides avenue for continued access and use of chloroquine in malaria treatment despite official discontinuation of its use [62, 63]. The low price coupled with ease of taking chloroquine compared to current artemisinin-based combinations, further influences continued non-prescribed use of chloroquine in malaria treatment [63]. Resistance selection in an area is an inevitable consequence of antimicrobial use especially when resistant organisms to the same medicine have already emerged in other areas [51, 61, 64]. It follows that reduction in extent of antimicrobial use would result in emergence of more susceptible micro-organisms [61]. This is exemplified by chloroquine in which withdrawal from malaria treatment resulted in the return of susceptible parasites [4, 65]. This pattern of re-emergence of chloroquine susceptible *P. falciparum* parasites across malaria affected regions has been observed in the current review. However, complete reversal to chloroquine sensitive parasites was not observed. This is likely due to continued drug pressure as chloroquine and related drugs are still accessed and used in the population [53]. The continued use of amodiaquine, a partner drug in artemisinin-based combinations for example has been shown to select for resistance to chloroquine [66].

Ursing et al. showed that even in presence of resistance, a change in dosing regimen restores chloroquine efficacy among *P. falciparum* parasites [67]. The study indicated that in vivo chloroquine failure rate can be decreased by giving the drug twice per day instead of once daily. Furthermore, doubling of chloroquine dose achieved 95% cure despite pre-existing parasite resistance with no observed increase in adverse events. In Guinea-Bissau, Ursing et al. [68] showed that doubling chloroquine dose helped stabilize spread of chloroquine resistance allele *Pfcrt* 76T. Pharmacodynamics modelling revealed that higher doses of chloroquine can eliminate resistant *P. falciparum* parasites [69]. Ginsburg [70] suggested that there is a finite concentration of chloroquine above which resistance will not develop. The hypothesized explanation for this suggestion is that the energy consumption required by the parasite to survive in presence of high chloroquine concentrations causes decrease in general fitness such that the parasites will not prevail [71]. A study by Nosten et al. [72] in Thailand also showed that if parasites develop resistance to a drug, combining that drug with an appropriate partner agent may in some cases effectively reduce drug pressure and allow for re-emergence of susceptible forms of the parasite. This helps inform current efforts considering re-introduction of chloroquine in malaria treatment. From recent evidence chloroquine can only be considered for re-introduction in malaria treatment in combination with other agents [73]. While a partner drug is sought to be combined with chloroquine, considerations should be made on the half- lives of potential agents with aim of having agents with similar half-lives to chloroquine [74, 75]. These observations are important in guiding efforts re-evaluating the potential emerging role of chloroquine in malaria treatment.

The review had some limitations, some of the articles did not report on all the review variables. Some variables were re-calculated from the data provided in the articles in addition to contacting authors to obtain more information and what was not established was reported as missing. Majority of the articles were from sub-Saharan African region however, since a wide article search criteria that was set a priori was applied, this is unlikely to affect the outcomes of the review.

## Conclusion

There is a general uneven distribution of decline without complete disappearance of chloroquine resistance alleles *Pfcrt* 76T and *Pfmdr*-*1* 86Y across malaria endemic regions following official discontinuation of chloroquine use. Implementation of complete withdrawal of chloroquine following policy change was not achieved in most countries thus its continued access and use could have contributed to persistence of resistance alleles in the population.

Evidence from the current review of prevalence of chloroquine resistance coupled with presence of highly efficacious artemisinin agents does not support re-introduction of chloroquine in malaria treatment in areas where chloroquine was previously used.

## Abbreviations

PRISMA: Preferred Reporting Items for Systematic reviews and Meta-Analysis
STROBE: Strengthening Reporting of Observational studies in Epidemiology
PROSPERO: International Prospective Register of Systematic Reviews
LMIC: low and middle income countries
STREGA: Strengthening the Reporting of Genetic Association Studies
